# Is the use of anthracyclines implicated in myocardial injury? Investigating the cardio modulatory effects of naringenin and apocynin in doxorubicin-induced cardiotoxicity in rats

**DOI:** 10.1016/j.toxrep.2025.102116

**Published:** 2025-08-21

**Authors:** Justin Atiang Beshel, Samuel Usoh Ukweni, Idara Asuquo Okon, Daniel Udofia Owu

**Affiliations:** a Department of Physiology, Faculty of Basic Medical Sciences, University of Calabar, Calabar, Nigeria; b Department of Physiology, Faculty of Biomedical Sciences, Kampala International University, Western Campus, Bushenyi, Uganda; c Department of Physiology, School of Medicine and Allied Health Sciences, Mwanza University, Tanzania

**Keywords:** Cardiac toxicity, Doxorubicin, Naringenin, Apocynin, Hypertension

## Abstract

Naringenin, a major flavonoid in oranges, grapefruit, tomato skin and apocynin a polyphenolic compound isolated from plants, such as *Apocynum cannabinum* are known to possess anti-oxidant, anti-inflammatory, and anti-cancer properties. Doxorubicin (DOX) is an antibiotic, effective in the treatment of cancer, but notorious for its propensity to cause cardiotoxicity. This study investigated the combined effects of naringenin and apocynin in DOX-induced cardiac toxicity. Thirty rats were randomly divided into five groups (n = 6) as follows: Normal Control (NC), DOX only, DOX+ naringenin, DOX + apocynin and DOX +naringenin + apocynin. DOX (2.5 mg/kg) was administered intraperitoneally, three times per week for two weeks (cumulative dose of 15 mg/kg). Naringenin (50 mg/kg/day) and apocynin (25 mg/kg/day) were administered orally. ECG measurements were carried out and heart homogenates were used to estimate cardiac inflammatory (IL-6, CRP), cardiac toxicity (CTnT, LDH, CKMB) and hypertensive (NO, ACE) markers. Histopathological examination of the heart was performed. Doxorubicin significantly altered the ECG with large T-wave, ST-elevation and wide QRS-complex. Results also showed significant changes in cardiac inflammatory and hypertensive biomarkers. Naringenin and apocynin treatment significantly attenuated the impact of doxorubicin on rats ECG, decreased biomarkers levels of cardiac inflammatory and hypertensive biomarkers. The cytoarchitecture of heart significantly improved in naringenin and apocynin treated groups, when compared to DOX only group. This study indicates that administration of naringenin and apocynin have cardioprotective ability and also ameliorated cardiotoxicity-induced by doxorubicin probably due to its anti-inflammatory and free radical scavenging properties.

## Highlights

• Doxorubicin a well-known anthracycline can cause cardiac toxicity.
• Cardiac toxicity is risk factor that can be mitigated by Naringenin and Apocynin.
• The ECG altered during cardiac injury can be improved by Naringenin and Apocynin.
• Combine effects of Naringenin and Apocynin can reduced the level of cardiac inframammary markers.
• Naringenin and Apocynin can restore the cytoarchitecture of the cardiac muscle.

## Graphical Abstract

**Figure:**
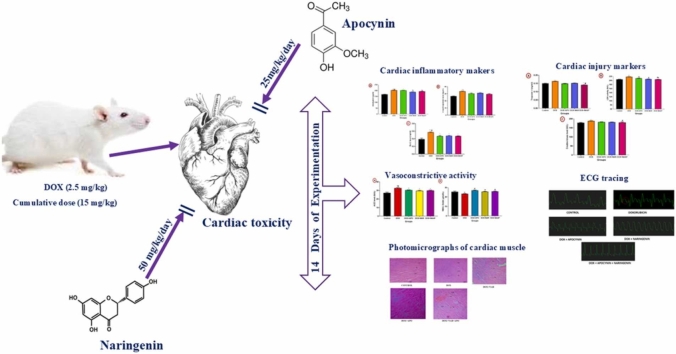

## 1 Introduction

Myocardial infarction (MI), informally known as “heart attack”, is one of the most common cardiovascular diseases (CVDs) prevalent worldwide [1]. It is caused by decreased or complete cessation of blood flow to a portion of the myocardium. This may be as a result of coronary artery occlusion, causing the myocardium to be deprived of oxygen [2]. Prolonged deprivation of oxygen supply to the myocardium can lead to myocardial cell death and necrosis. Risk factors include; smoking, abnormal lipid profile, hypertension, diabetes mellitus, abdominal obesity, psychosocial factors such as depression, unhealthy diet, lack of physical activity, excessive alcohol intake and cardiotoxicity [3]. Cardiovascular diseases are the leading causes of death globally and the second leading causes of death in Africa. An estimated 17.9 million people died from CDVs in 2019, representing 32 % of all global deaths. Of these deaths, 85 % were due to heart attack and stroke [4]. Treatment using a family of antibiotics known as anthracyclines, which includes; doxorubicin, daunorubicin, epirubicin and idarubicin, (used to treat solid tumors such as; ovary, breast, stomach, brain, and gastrointestinal tumors and hematological malignancies like lymphoma and pediatric leukemia) [5], are becoming a major cause for concern due to their propensity towards cardiovascular diseases which has become a leading cause of morbidity and mortality among cancer survivors receiving anthracycline therapy [6].

Cardiotoxicity induced by doxorubicin (DOX), a highly effective antibiotic used in the treatment of cancer, is becoming an important health problem for oncological patients [7]. Previous studies have shown that the use of doxorubicin is associated with cumulative asymptomatic cardiotoxicity that presents even after many years of cessation of chemotherapy which may lead to cardiac dysfunction and cardiomyopathic changes, resulting in severe heart failure and death [8], [9]. This is because, doxorubicin-induced cardiotoxicity is associated with an alteration of several cellular events, including increased generation of reactive oxygen species (ROS), lipid peroxidation, mitochondrial dysfunction, and calcium overload, leading to cellular oxidative stress as well as activation of various cell death pathways such as apoptosis and necrosis [10].

Amongst the plethora of agents investigated for cardioprotective effects, study on the beneficial effects of naringenin and apocynin is of special interest and of clinical relevance due to their well-researched anti-oxidant activities. Naringenin (5,7-dihydroxy-2-(4-hydroxyphenyl) chroman-4-one) is a major natural flavonoid found in oranges, grapefruit and tomato skin [11]. It has anti-oxidant and anti- inflammatory properties, thereby making it a prospective therapy with great potentials in the treatment of various disorders such as neurodegenerative, cardiovascular, diabetes mellitus and malignancies [12]. The pharmacological activities associated with naringenin have been attributed to its ability to suppress oxidative stress by scavenging free radicals generated during various basal metabolic conditions [13].

Apocynin (4-hydroxy-3-methoxy-acetophenone) also known as acetovanillone is a natural polyphenolic compound isolated from a variety of plant sources, including *Apocynum cannabinum* (Indian hemp) and *Picrorhiza kurroa* [14]. It has multiple pharmacological effects, such as anti-oxidant, anti-inflammation, and anticancer effects [15]. Study has shown that there are limitations in other medications such as dexrazoxane used in treatment and management of anthracycline-induced cardiotoxicity [16]. For instance, the cardioprotective effects of dexrazoxane does not significantly mitigate the overall survival or progression-free survival [17]. Other clinical trial study suggests that dexrazoxane provides about 76 % risk reduction when administered with doxorubicin [16]. However, naringin has been proven to completely reduce myocardial hypertrophy in an isolated heart due to its high flavonoid content [18]. Additionally, the primary bioactive flavonoid in citrus fruits, naringenin, has demonstrated promise as an alternative to cancer treatment [19]. Also, apocynin derivatives can interrupt intracellular signaling that could result in decrease migration of breast and prostate cancer cells [20] while reducing cardiotoxicity [21].

Apart from examining the electrical activities of the heart, many cardio-modulatory biomarkers are used to assess the integrity and functionality of the myocardium in disease condition. These include myocardial infarction parameters, hypertensive and cardio-inflammatory markers. Previously documented study has confirmed that these parameters can serve as a diagnostic tool in managing cardiac toxicity [22]. Additionally, there is a positive correlation between cardiac toxicity, hypertension, cardiac inflammation and myocardial infarction in healthy subjects [23]. Due to emerging scientific reports on the adverse effects of DOX, we carried out this study to investigate the cardio-modulatory effect of naringenin and apocynin in DOX-induced cardiotoxicity in rats.

Unlike other antioxidant agents with just an antioxidant potential, naringenin, a citrus flavanone, is a potent antioxidant with anti-inflammatory, anti-cancer, and neuroprotective properties, acting on multiple levels of oxidative stress [24]. Apocynin on the other hand is a special kind of antioxidant that inhibits NADPH oxidase (NOX), which directly targets sources of reactive oxygen species (ROS) [25]. These unique qualities make the basis for the choice of these two antioxidant agents for this study. Although studies have been conducted on antioxidant properties of naringenin and apocynin separately, no work has been done on their combined effects in anthracycline-induced cardiotoxicity. Our study seeks to bridge this gap by investigating the combined effects of naringenin and apocynin in DOX-induced cardiotoxicity and to explore possible synergistic potential of the two unique plant products in mitigating cardiotoxicity caused by anthracyclines with special interest in doxorubicin.

## 2 Methods

### 2.1 Drugs and chemicals

Doxorubicin was purchased from Zydus Lifesciences Ltd. Thane, India while naringenin was purchased from Sigma-Aldrich Company, St Louis, Mo USA. Apocynin was bought from EMD Millipore Corp, MA, USA and ketamine and xylazine were purchased from Swiss Parenterals Ltd, Gujarat, India. Cardiac troponin, creatine kinase, lactate dehydrogenase enzyme kits were purchased from Cusabio Biotech Co. (China). These drugs and chemicals used were of analytical grade.

### 2.2 Use and care of animals

Thirty (30) healthy, male Wistar rats aged seven weeks weighing 160–200 g were purchased from the animal house of the Faculty of Basic Medical Sciences, University of Calabar. Every animal procedure complies with the guidelines established by the animal Care and Use Committee (ACUC), the Animal Welfare Act, and the Guide for the Care and Use of Laboratory Animals. The study was approved (290PHY3624) by the Animal Research Ethics Committee of Faculty of Basic Medical Sciences, University of Calabar.

### 2.3 Study design

The thirty rats were randomly divided into five groups (n = 6) as follows: Group I: Normal Control (NC) that received 0.1 ml of normal saline intraperitoneally, three times per week for two weeks. Group II: Doxorubicin only (DOX) received 2.5 mg/kg doxorubicin intraperitoneally, three times per week for two weeks. Group III: Doxorubicin + Naringenin (DOX + NAR) received 50 mg/kg/day of naringenin orally and 2.5 mg/kg of doxorubicin intraperitoneally, three times per week for two weeks. Group IV: Doxorubicin + Apocynin (DOX + APO) received 25 mg/kg/day of apocynin orally and 2.5 mg/kg of doxorubicin intraperitoneally, three times per week for two weeks. Group V: Doxorubicin + Naringenin + Apocynin (DOX + NAR + APO) received 50 mg/kg/day of naringenin orally, 25 mg/kg/day of apocynin orally and 2.5 mg/kg of doxorubicin intraperitoneally, three times per week for two weeks. Base on efficacy studies, induction of cardiotoxicity using DOX was followed as previously reported by Naderi et al. [26], while the doses of naringenin and apocynin were chosen base on the determination of LD_50_ and efficacy as previously reported [27], [28].

### 2.4 Induction of cardiotoxicity

Cardiotoxicity was induced by administering a total cumulative dose of doxorubicin (15 mg/kg) intraperitoneally, within a short time of two weeks. This dose was selected on the basis of previously reported studies and has been shown to be sufficient to induce acute cardiac damage in rats [29].

### 2.5 Administration of naringenin and apocynin

Naringenin was prepared by dissolving 100 mg in 10 ml of normal saline to form a stock solution. It was administered at a dose of 50 mg/kg/day orally using an orogastric tube for 14 days. Naringenin dose was selected based on earlier experimental studies to determine its LD_50_ value in rats, and 100 mg/kg of naringenin was considered both safe and effective as previously reported [30]. Apocynin was prepared by dissolving 50 mg in 10 ml to obtain a stock solution. From the stock solution, it was administered at a dose of 25 mg/kg via oral gavage for 14 days [28].

### 2.6 Recording of electrocardiogram (ECG) of rats

The ECG measurement in rats was done as previously documented by Lin et al. [31]**.** Electrodes were plugged into the ECG monitoring machine (Heart and Brain SpickerBox; Backyard Brains, Inc. 308 1/2 S. State Street, Suite 35, Ann Arbor, MI 48104) which was connected through the USB to a computer (Intel Core 2 Duo) running the Spike Recorder software. ECG signals were captured with the software and data logged to disk as a file in the sound (wave) format. Recordings were initiated when stable and consistent signal waveforms were observed on the computer monitor and each recording session lasted for two minutes.

### 2.7 Measurement of body weight and organ weight

The body weight of the rats was recorded on day 1, 3,5,7,10,12 and 14 using a digital weighing balance. At the end of experiment, the heart was carefully harvested, cleared of connective tissues, weighed using a sensitive electronic weighing balance. The relative organ weight was calculated using the formula: Relative organ weight (%) = [Absolute organ weight (g) / Final body weight (g)] x 100.

### 2.8 Collection of tissue

At the end of the 14 days, the animals were anaesthetized by intraperitoneal administration of ketamine (60 mg/kg) and xylazine (6 mg/kg) and blood was collected via cardiac puncture with a 5 ml sterile syringe into plain sample bottles for biochemical analysis. The blood samples were centrifuged at 1500 g per minute for 10 min to separate the serum. The sera were collected and stored at −20°C till subsequent use for biochemical analysis.

The hearts were excised, trimmed of connective tissues, cleaned and weighed in a balance. A section of the heart was immersed in phosphate buffered saline (PBS, pH 7.4) and homogenized in ice-cold PBS to form 10 % of the heart homogenate. Thereafter, the homogenate was centrifuged using axygen ™ refrigerated centrifuge (China) at 1000 rpm for 10 min. The supernatant was collected and stored in fresh sample tubes at −4°C until required. The remaining part of the heart was used for histopathological examination.

### 2.9 Determination of cardiac inflammatory biomarkers

Interleukin 6 assay was done using rat Elisa kit following the manufacturer’s instruction. C-reactive protein (CRP) (Biosystems S.A spain) levels were measured using competitive enzyme immunoassay technique based on the manufacturer’s protocol [32].

### 2.10 Determination of biomarkers of cardiac toxicity

Biomarkers of cardiac toxicity which include cardiac troponin, lactate dehydrogenase and creatine kinase were determine following appropriate methods. Rat cardiac troponin level was assay using Cusabo Elisa Kits. The Lactate dehydrogenase and CK-MB activity were measured using Cusabio Elisa test kit as previously reported [33].

### 2.11 Determination of hypertensive markers

Angiotensin converting enzyme activity was assay using rat ACE activity kit. Nitric oxide was assay using the colorimetric assay kit [34]. Nitric Oxide Colorimetric test provides an accurate, convenient measure of total nitrate/nitrite in a simple two-step process.

### 2.12 Histopathological examination of the heart

The hearts were fixed in 10 % formalin for 48 h. Thereafter, the tissues were cut longitudinally, placed in a cassette, and processed using an automated tissue processor (Leica, Germany). The process involved sequential immersion of the cassettes in 80 % ethanol for 1 h, two changes in 95 % ethanol for 1 h each, three changes in absolute ethanol for 1 h each, three changes in xylene for 1 h each, and two changes in warm (60 ± 2 °C) paraffin for 2 h each. Thereafter, the tissues were embedded in blocks of paraffin and stored at −20°C until use. Sections of thickness 5 µm were cut using a microtome, placed in a water bath (42 ± 1 °C), fetched on to a glass slide, and stained using hematoxylin and eosin (H & E) technique [33].

The histological changes of the slides were examined by two pathologist who were not privy to the experiment to avoid bias. The scoring of cardiac tissue abnormalities was based on the modified method reported by Patintinga et al. [35] as little or no cardiac abnormality (0), mild cardiac abnormality such as few inflammatory cells (1), moderate cardiac abnormality with few necrotic zones (2) and severe cardiac abnormality with many inflammatory cells and severe necrosis (3).

### 2.13 Statistical analysis

Results were expressed as mean ± standard error of mean (SEM). Data for the results were analyzed by one-way analysis of variance (ANOVA), followed by Tukey’s post-test using GraphPad prisms software version 9.0 (GraphPad Software, San Diego, California, USA) and p-value of less than 0.05 was considered statistically significant.

## 3 Results

### 3.1 Doxorubicin-induced weight loss

**Fig. 1:**
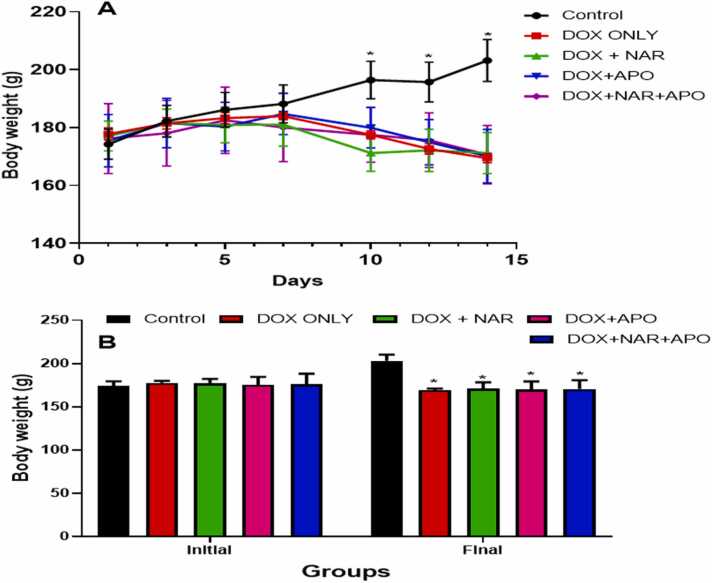
(A-B). Body weight changes and final body weights in rats treated with doxorubicin, naringenin and apocynin. Fig. 1A. Daily body weight change. Fig. 1B. Final body weight change. *= p < 0.05 compared with control.

The mean body weight of animals in all the groups was determined before the start of the experiment. No significant change in the initial mean body weight was observed between the groups. However, the final mean body weight showed a significant (p < 0.05) decrease in DOX only, DOX+NAR, DOX+APO and DOX+NAR+APO, when compared to control (see Fig. 1A-B).

**Fig. 2:**
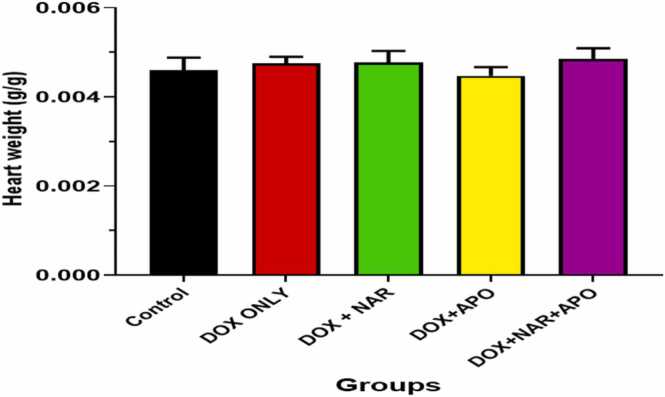
Relative heart weight in control and experimental groups treated with naringenin and apocynin**.** No significant difference.

Changes in relative heart weight of the experimental animals were observed (see Fig. 2). At the end of the study, the mean relative heart weight showed no significant difference between the experimental groups when compared to the control.

### 3.2 Naringenin and apocynin reversed abnormal ECG tracings in Dox-induced cardiac toxicity

**Fig. 3:**
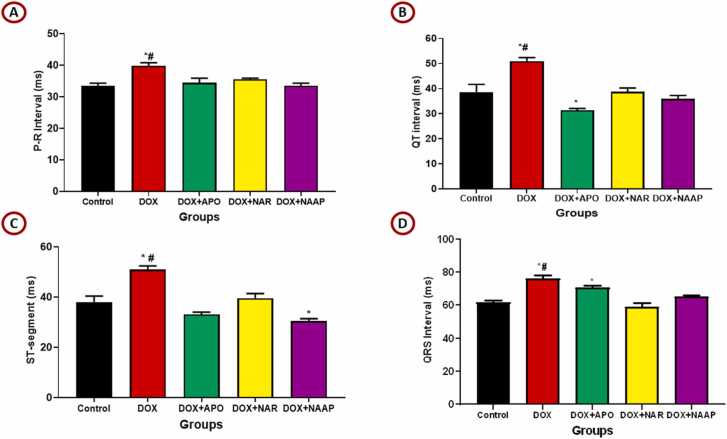

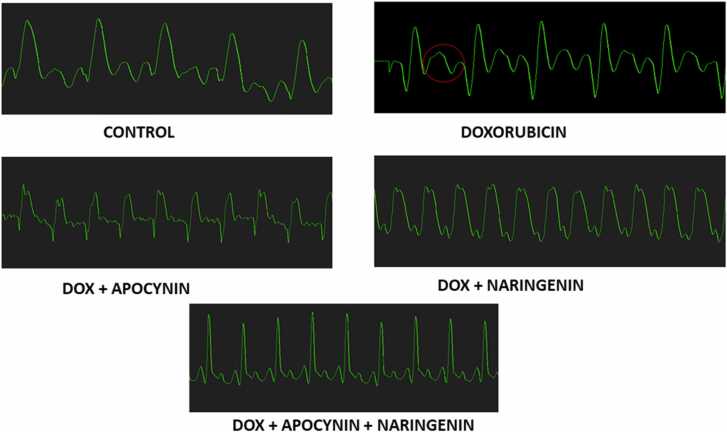
(A-D)**.** ECG waves of the different experimental groups. Fig. 3A. (P-R Interval), Fig. 3B. (QT interval), Fig. 3C. (ST segment), Fig. 3D (QRS interval). *= p < 0.05 compared with control; # = p < 0.05 compared with other doxorubicin-treated groups. E. Representative ECG tracing in control and groups treated with naringenin and apocynin following myocardial infarction. Red cycle shows the T-wave elevation.

ECG tracing (Fig. 3A-D) showed normal pattern in the control and all the other studied groups except the doxorubicin treated group. Rats in the doxorubicin treated group revealed ECG alterations including abnormally elevated T waves as compared to the control group. On the other hand, the treatment with naringenin as well as apocynin ameliorated these alterations.

#### 3.2.1 P-R interval

The mean P-R interval for the various experimental groups is represented (see Fig. 3A). There was a significant (p < 0.05) increase in the P-R interval value of DOX only compared to control, DOX + NAR, DOX + APO and DOX + NAR + APO groups respectively.

#### 3.2.2 QT- interval

Waves deflection in the QT-interval (Fig. 3B) showed a significant (p < 0.05) increase in the QT interval value of DOX only compared to control, DOX + NAR, DOX + APO and DOX + NAR + APO groups respectively.

#### 3.2.3 ST-segment

The result for ST-segment (Fig. 3C) also showed a significant (p < 0.05) increase in the ST-segment value of DOX only compared to control, DOX + NAR, DOX + APO and DOX + NAR + APO groups respectively. Additionally, combined treatment with naringenin and apocynin significantly (p < 0.05) decreased the ST-segment elevation as observed in DOX + NAR + APO when compared to control and DOX + NAR groups.

#### 3.2.4 QRS-complex

The mean QRS-Complex for the various experimental groups is represented (see Fig. 3D). There was a significant (p < 0.05) increase in the QRS-complex value of DOX only compared to control, DOX + NAR and DOX + NAR + APO groups respectively.

The representation of these changes in ECG waves, is presented in Fig. 3E.

### 3.3 Treatment with naringenin and apocynin augmented the abnormal increase in cardiac injury makers

**Fig. 4:**
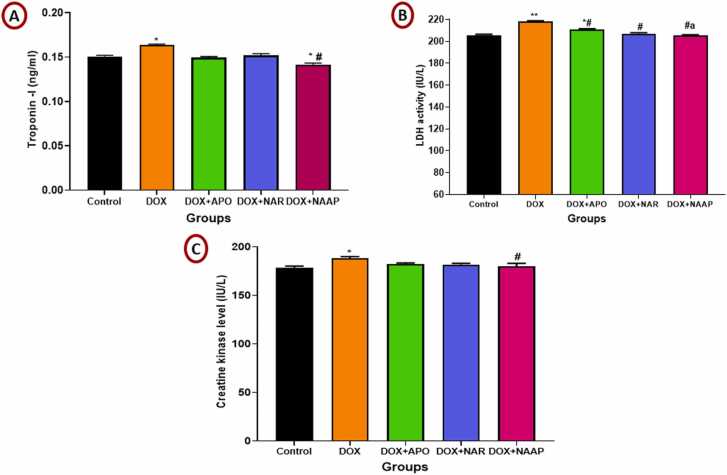
(A-C). Treatment with naringenin and apocynin augmented the abnormal increase in cardiac injury makers. Fig. 4A. (Cardiac troponin I), Fig. 4B. (Lactate dehydrogenase), Fig. 4C. (Creatine kinase). *= p < 0.05 versus control group; # =p < 0.05 compared with DOX treated groups; a = p < 0.05 compared with DOX+ Apocynin group.

Result for assessment of the levels of cardiac injury caused by Dox is presented (see Fig. 4A-C). These injury markers were cardiac troponin-I (CTnI), lactate dehydrogenase (LDH) and creatine kinase muscle and brain (CKMB).

The mean value for Cardiac troponin-I is represented in Fig. 4A. There was a significant (p < 0.05) increase in the cardiac troponin-I in DOX only compared to control, DOX + APO, DOX + NAR and DOX + NAR + APO groups. However, combined treatment with naringenin and apocynin after inducing cardiac toxicity showed a significant decrease (p < 0.05) in cardiac troponin-I when compared to control, DOX + APO and DOX + NAR groups.

The cardiac LDH level was significantly (p < 0.01) higher in DOX only compared to control and (p < 0.05) increase when compared to DOX + APO, DOX + NAR and DOX + NAR + APO groups respectively. DOX + APO also showed a significant increase (p < 0.05) in lactate dehydrogenase when compared to control, DOX + NAR and DOX + APO + NAR groups. While DOX + APO, DOX + NAR and DOX + APO + NAR showed significant decrease (p < 0.05) when compared to DOX only group. DOX + APO + NAR also showed significant decrease (p < 0.05) when compared to DOX + APO group (see Fig. 4B)

The mean value for CKMB level is represented (see Fig. 4C). There was a significant (p < 0.05) increase in CKMB level of DOX only when compared to control and DOX + NAR + APO groups

### 3.4 Naringenin and apocynin reduced abnormal increase in cardiac inflammatory response in Dox-induced cardiac toxicity

**Fig. 5:**
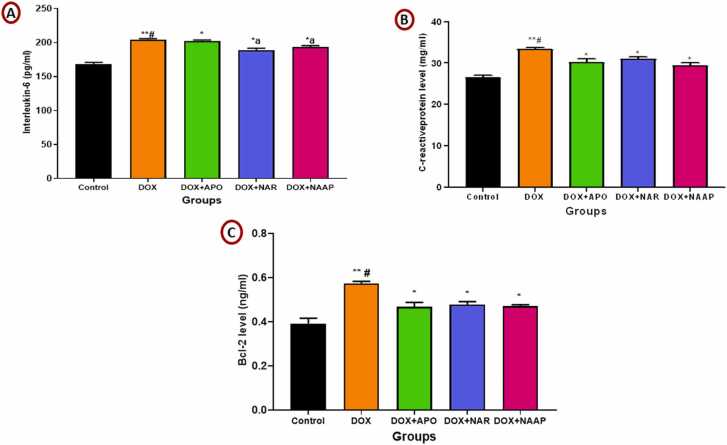
(A-C). Naringenin and apocynin reduced abnormal increase in cardiac inflammatory response in Dox-induced cardiac toxicity. Fig. 5A. (Interleukin-6), Fig. 5B. (C-reactive protein), Fig. 5C (BCL-2). *= p < 0.05 versus control group; # =p < 0.05 compared with DOX treated groups; a = p < 0.05 compared with DOX+ Apocynin group.

The mean value for Interleukin-6 is represented in Fig. 5A. There was a significant (p < 0.01) increase in the cardiac Interleukin-6 of DOX only compared to control and (p < 0.05) increase when compared to DOX + NAR and DOX + NAR + APO groups respectively. DOX + APO also showed a significant increase (p < 0.05) in Interleukin-6 when compared to control, DOX + NAR and DOX + APO + NAR groups. While DOX + NAR and DOX + APO + NAR showed significant increase (p < 0.05) when compared to control.

The mean value for C-reactive protein level is represented in Fig. 5B. There was a significant (p < 0.01) increase in C-reactive protein level of DOX only compared to control and (p < 0.05) increase when compared to DOX + APO, DOX + NAR and DOX + NAR + APO groups respectively. However, C-reactive protein levels were significantly (p < 0.05) higher in DOX + APO, DOX + NAR and DOX + NAR + APO, when compared to control.

The mean value for Bcl-2 level is represented in Fig. 5C. There was a significant (p < 0.01) increase in Bcl-2 level of DOX only compared to control and (p < 0.05) increase when compared to DOX + APO, DOX + NAR and DOX + NAR + APO groups respectively. However, Bcl-2 levels were significantly (p < 0.05) higher in DOX + APO, DOX + NAR and DOX + NAR + APO, when compared to control.

### 3.5 Treatment with naringenin and apocynin reversed vasoconstrictive activity in Dox-induced cardiac toxicity

**Fig. 6:**
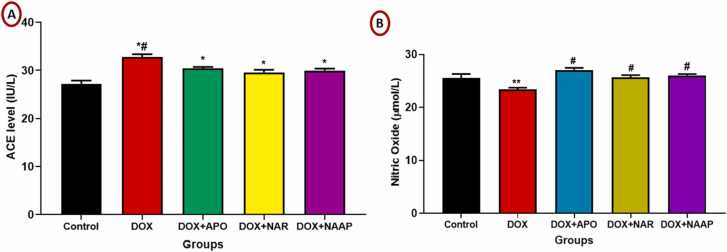
(A-B). Treatment with naringenin and apocynin reversed vasoconstrictive activity in Dox-induced cardiac toxicity. Fig. 6A (Angiotensin converting enzymes activity), Fig. 6B. (Nitric oxide). *= p < 0.05 compared with control; # = p < 0.05 compared with DOX-treated group with apocynin and naringenin.

The mean value of ACE activity is represented in Fig. 6A. There was a significant (p < 0.05) increase in ACE level of DOX only compared to control and when compared with DOX + APO, DOX + NAR and DOX + NAR + APO groups respectively. However, ACE levels of DOX + APO, DOX + NAR and DOX + NAR + APO groups were significantly higher (p < 0.05), when compared with control.

The mean value of the nitric oxide level was measured and is represented in Fig. 6B. There was a significant (p < 0.01) decrease in nitric oxide level of DOX only when compared to control. However, nitric oxide levels of DOX + APO, DOX + NAR and DOX + NAR + APO groups were significantly higher (p < 0.05), when compared with DOX only group.

### 3.6 Naringenin and apocynin abates histopathological changes in cardiac muscles

**Fig. 7:**
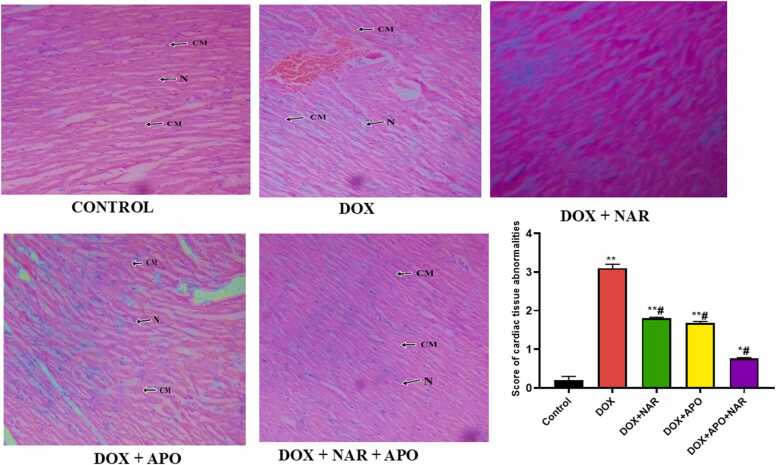
Photomicrographs of a cross section of the cardiac muscles in different experimental groups Magnification – (x 100), CM-Cardiac muscle, N-Nucleus. *= p < 0.05 compared with control; # = p < 0.05 compared with DOX-treated group with apocynin and naringenin.

Histological changes in the cardiac tissues of doxorubicin, apocynin and naringenin treated rats is represented (see Fig. 7). Section of the cardiac muscle in the control group shows prominent cardiac myocytes and connective tissue. The cardiac myocytes have prominent nuclei and sparse amount of fibro-collagenous tissue with attached elongated fibroblast. In the DOX only, the cardiac myocytes are hypertrophied with prominent nuclei. The intervening fibro-collagenous tissue is scanty with attached elongated fibroblast. However, in DOX + NAR treated rats, section of the cardiac muscle shows prominent hypertrophic cardiac myocytes and fibro-collagenous connective tissue. The cardiac myocytes have prominent nuclei. The muscle shows distinct intercalated disc and prominent interdigitation. The nuclei of the cells appear ovoid and enlarged. In the DOX + APO**,** the cardiac muscle shows hypertrophied cardiac myocytes with poorly outlined centrally located oval nuclei. The interdigitation is prominent and fibro-collagenous connective tissue is scanty with attached fibroblast. The cardiac muscles in the combined treated group (DOX + NAR + APO**)** showed mildly hypertrophied cardiac myocytes with poorly outlined centrally located oval nuclei. The interdigitation is prominent and fibro-collagenous connective tissue is scanty with attached fibroblast. There is mild cardiac rupture with mild haemorrhage.

Additionally, after examining the cardiac tissue, the scoring of cardiac tissue abnormalities revealed higher abnormalities such as myocardial necrosis, inflammatory cell infiltration and bleeding between myocardial fibres in DOX only group (P < 0.05) when compared to the control and treated groups. However, a significant (P < 0.05) decrease was observed in DOX + NAR +APO treated group when compared with DOX + NAR and DOX + APO.

## 4 Discussion

The prevalence of cardiovascular complications associated with anthracyclines treatments such as doxorubicin has increased in cancer survivors accounting for about 33 % deaths due to chemotherapy associated heart failure [36]. The modulatory effect of naringenin and apocynin co-administration in doxorubicin-induced cardiotoxicity was studied in Wistar rats.

The changes observed in electrical activities (ECG) of the heart reveals alteration in cardiac functions after 14 days of doxorubicin administration characterised by an elevated T-waves, increased P-R interval, prolonged Q-T interval, increased S-T segment and enlarged QRS complex. DOX is reported to denature the cell membrane and alter membrane function by causing lipid peroxidation [37]. This alters ionic composition of the cell that may be responsible the ECG changes and cardiac rhythm observed in this study. Hyperkalaemia is a common cause of large T-waves in ECG [38] and is an early sign of ST-elevation used for diagnosis of myocardial infarction and ventricular hypertrophy [39]. P-R interval reflects the distribution of depolarization from the atrium to the ventricles and is used to atrioventricular blocks [40]. The increase in P-R interval observed in our study supports the previous research that reported an increase in P-R interval caused by doxorubicin administration [41] and is in line with our recent finding that doxorubicin impedes transmission of electrical impulses in the myocardium [38].

The Q-T interval represents the time of the ventricular cardiomyocyte depolarization and repolarization. Prolonged duration of this interval indicates an intrinsic heart disease or toxic effects of exogenous substances and it is a useful indicator of the drug-induced cardiotoxicity. The present result showed an increase in the Q-T interval by DOX and confirms a previous investigation that reported a similar finding caused by doxorubicin [42]. The S-T segment is the time between the end of the QRS complex and the commencement of the T-wave and is a diagnostic value for myocardial infarction and ischemia. Administration of doxorubicin prolonged this S-T segment and is in line with a previous report [43].

The QRS complex represents ventricular rhythms, and disruptions reflect cardiac insufficiency, myocardial ischemia, and ventricular blocks [42]. Wide QRS complexes were observed in DOX-only treated group in this study. This can be attributed to the down-regulation of connexin 43 (Cx43), a gap junction protein that is extensively expressed in ventricular myocytes and plays an essential role in electrical signal transmission between myocytes [42]. Previous report has also reported that doxorubicin induced a widened QRS that was associated with the down-regulation of this gap junction protein [44] that can contribute to the arrhythmias and other heart-related complications that can occur with doxorubicin therapy.

Naringenin and apocynin treatment significantly mitigated these acute changes in ECG. The protective effect of naringenin may be mediated through its ability to decrease membrane fluidity and therefore prevent leakages of across the membrane [45]. A study has reported that naringenin treatment significantly reduced doxorubicin-induced lipid and protein peroxidation and improved endothelial function through the production of nitric oxide [46], and facilitated endothelial cell vasodilatation in the heart vessels [47]. Furthermore, naringenin inhibits the L-type calcium channels, which plays a role in repolarization of the membrane, thus causing relaxation, reducing apoptosis and promoting cell viability [48], [49], [50]. Apocynin on the other hand induces NO generation, thereby improving blood flow and protecting the heart [51], and opens the mitochondrial ATP-dependent K^+^ channel, thereby improving contractile function [52]. Apocynin caused complete block of calcium release from the sarcoplasmic reticulum by limiting Ca^2+^ release to acidic stores such as lysosome during myocardial ischemia [51]. These mechanisms could have been responsible for the amelioration of ECG changes caused by doxorubicin by these agents.

This study also examined the effect of naringenin and apocynin on cardiac biomarkers namely cardiac troponin-I, lactate dehydrogenase (LDH), creatine kinase (CK). These markers exist in myocardial tissues and are elevated in blood during myocardial infarction [53]. Results showed a significantly higher levels of these cardiac biomarkers in DOX-only rats when compared to control. This is consistent with previous results that reported increased enzyme activities of CK, LDH [54] and cardiac troponin-I in DOX-only treated rats [55]. This might be as a result of excessive production of free radicals and lipid peroxides that caused leakage of these cytosolic enzymes due to cell membrane damage. The decline on cardiac troponin-I, LDH and creatine kinase when compared to DOX-only group is an indication of the ameliorative effect of naringenin and apocynin. Naringenin treated groups showed significantly lower levels when compared to their apocynin counterparts. This is because naringenin scavenges free radicals and inhibits the hydrogen peroxide induced lipid peroxidation [55]. Conversely, apocynin generates NO that is a strong scavenger for ROS thus reducing ROS availability thereby exhibiting cardioprotective effect [51].

Inflammatory markers such as interleukin-6 and C-reactive protein, and apoptotic biomarker, B-cell lymphoma-2 were also measured. Inflammatory markers are indicative of injury to the heart muscle following cardiotoxicity. There was a significant increase in the levels of these markers in all doxorubicin-treated groups when compared to control, an indication of doxorubicin’s ability to cause myocardial injury. Elevated levels of C-reactive protein in doxorubicin-treatment animals have been reported in previous study [56]. However, administration of naringenin and apocynin significantly reduced the level of these markers as compared to the DOX only group, which is an indication of its ameliorative effect on myocardial injury, oxidative stress and apoptosis. Naringenin have been reported to reduce the expression of signalling molecules such as IL-6, IL-8, inducible nitric oxide synthase (iNOS), and nuclear erythroid-related factor (Nrf2) associated with heart injury [57]. This effect of naringenin is also in line with another study that naringenin inhibit inflammatory cytokine action and attenuate inflammation via the inhibition of NF_k_B and regulation of leukocyte adhesion [47] Naringenin also increases the expression of Nrf-2 as an antioxidant modulator to produce more antioxidants. Apocynin treatment was reported to cause a significant decrease in the levels of inflammatory biomarker TNF-α, IL-1β, and IL-6 [51]. Conversely, there was increased anti-apoptotic marker Bcl-2 in DOX-only group when compared with control. Increased Bcl-2 expression can protect against cardiotoxicity caused by doxorubicin as previous studies show that doxorubicin exposure increases anti-apoptotic protein expression such as Bcl-2, followed by decreased expression [58], [59].

The ACE is a key enzyme involved in the renin-angiotensin-aldosterone system involved in pathophysiology of many diseases such as arterial hypertension, heart failure, and even cancer [60]. ACE activity was increased in DOX-only group when compared with the control. This rise could be attributed to infiltration of the myocardium by macrophages. It has been reported that doxorubicin can cause about 2.3fold increase in cardiac ACE activity after treatment [61], [62].

Histopathological examination of the heart, kidney and liver tissues obtained from control animals exhibited clear integrity of the fibres without infarction. The doxorubicin-treated animals showed disruption of several subcellular elements such as loss of fibro collagenous tissue, vacuolization, haemorrhage and hypertrophy which may be due to protein and lipid peroxidation, disturbance of the energy metabolism pathways of the cells and poor substrate utilization, in accordance with reports from previous studies [63]. Treatment with naringenin and apocynin were able to mitigate these changes as reflected in the injury score indexes which may be attributed to their anti-oxidant properties [46], [51].

Significant reduction in body weight was observed in all doxorubicin-treated animals. Previous studies have reported loss in body weight in animals administered with doxorubicin, which could be due to mucositis and diminished protein synthesis following its effect on the liver [64], [65]. Additionally, administration of DOX can activate the ubiquitin-proteasome pathway, resulting in reduced protein synthesis and body weight [66], [67]. Although an increase in body weight was observed, treatment with naringenin and apocynin were unable to significantly recover this weight loss probably due to the short duration of this study [67]. No marked change was observed in the relative heart weight, which could also predict the duration/dosage deteriorative effects of doxorubicin on organ weight [68].

### 4.1 Study limitation

Limitation of this study include lack of detailed mechanistic approach on the effect of Dox in cardiac toxicity and the dose dependent effects of naringenin and apocynin. Additionally, the short duration of this study may also influence our result. Future study should focus on this to ascertained the ameliorative potential of naringenin and apocynin in Dox-induced cardiotoxicity.

## 5 Conclusion

In conclusion, the administration of naringenin and apocynin has cardioprotective ability and is able to ameliorate cardiotoxicity induced by doxorubicin probably due to its intervention in the ROS and NO generating pathway.

## CRediT authorship contribution statement

**Daniel Udofia Owu:** Writing – review & editing, Project administration, Conceptualization, Formal analysis, Supervision. **Samuel Usoh Ukweni:** Resources, Investigation, Writing – original draft, Methodology, Data curation. **Justin Atiang Beshel:** Supervision, Investigation, Methodology, Conceptualization. **Idara Asuquo Okon:** Writing – review & editing, Software, Data curation, Writing – original draft, Formal analysis.

## Ethical approval

The study was approved (290PHY3624) by the Animal Research Ethics Committee of Faculty of Basic Medical Sciences, University of Calabar.

## Funding

This study received no funding.

## Declaration of Competing Interest

The authors declare that they have no known competing financial interests or personal relationships that could have appeared to influence the work reported in this paper.

## Data Availability

Data will be made available on request.
